# Bond strength between titanium and polymer-based materials adhesively cemented

**DOI:** 10.1080/26415275.2021.1937182

**Published:** 2021-06-20

**Authors:** Camilla Johansson, Aleksandra Håkansson, Evaggelia Papia

**Affiliations:** Department of Materials Science and Technology, Faculty of Odontology, Malmö University, Malmö, Sweden

**Keywords:** Adhesive cementation, bond strength, polymer-based materials, titanium base implant-supported dental prosthesis

## Abstract

The aim was to evaluate the bond strength between titanium and polymer-based materials for prosthetic restorations, cemented with different adhesive cement systems. Eight groups with 13 specimens in each group were included. Each specimen consisted of two parts: a cylinder of titanium resembling a titanium base, and a cylinder of one of two polymer-based materials Micro Filled Hybrid (MFH) or Telio CAD and cemented with one of four adhesive cement systems, namely Multilink Hybrid Abutment, Panavia V5, RelyX Ultimate and G-Cem LinkAce. The titanium was sandblasted with 50 µm Al_2_O_3_ and treated according to each cement manufacturer's recommendations. The polymer-based materials were pre-treated according to the manufacturer's instructions including sandblasting for MFH. After cementation, the groups were water stored for one day before thermocycling: 5000 cycles in 5–55 °C. A shear bond strength test was performed (crosshead speed 0.5 mm/min) and data was analysed with one-way ANOVA, Tukey's test. Telio CAD cemented with Panavia V5 and G-Cem LinkAce showed significantly lower bond strength compared to all other groups, due to spontaneous debonding. The highest numerical bond strength was found in the group of MFH cemented with RelyX Ultimate or with G-Cem LinkAce. Generally, the Telio CAD groups showed lower bond strength values than the MFH groups. The conclusions are that pre-treatment methods and choice of cement system are of importance for polymer-based materials for prosthetic restorations. The bond strength is adequate for provisional cementation irrespective of cement system when pre-treating by sandblasting, but cement dependent without sandblasting.

## Introduction

Following Professor Per Ingvar Brånemark's discovery and his first dental implant operation in 1965, patient treatments using titanium implants have been proven to be successful for replacing missing teeth [1,2]. The fixture replaces the root of a tooth, hence the jaw bone is loaded in a similar way as with a vital tooth, reducing the risk of bone loss [3,4]. For bone-level implants, an abutment, either separate or integrated with the restoration, is screw-connected to the fixture, and the prosthetic restoration is cemented or screw-retained to the abutment [5–7]. The abutment can be made either entirely in titanium or zirconia, preferably with a titanium base [6]. The abutment and base can be designed with different heights, geometries and surface roughness [8,9], affecting the retention of the prosthetic restoration, which is commonly made of ceramics or metal ceramics [6,7,10,11]. In addition to these materials, polymers such as polymethyl methacrylate (PMMA) have begun to be used [6,12,13]. PMMA is most suitable for temporary fixed dental prostheses (FDPs) due to its inferior mechanical properties and is usually adhesively cemented extraorally directly to a titanium base, allowing excess cement to be easily removed [7,11,14,15]. A correctly designed temporary crown, immediately placed, facilitates shaping of the emergence profile, can reduce the number of technical complications and serves as model when designing the permanent restoration [15–18]. Temporary restorations require a sufficient and stable bond even though they are mainly indicated for short- or long-term use and eventually have to be removed.

Although PMMA is a commonly used material for temporary crowns and the use of titanium bases is increasing, the literature on implant-supported PMMA temporary restorations with titanium bases is limited [15,17,19]. Rodrigues et al. concluded that temporary crowns made of PMMA with a cobalt chromium base can be suitable for up to six months, regarding survival probability [19]. In a study that evaluated polymer-infiltrated ceramic network restorations with titanium bases, Tribst et al. found the highest stress concentrations in the emergence profile area and emphasized the need for further studies investigating the interface and bond strength between cement, restoration and titanium base [17].

For a successful outcome, cemented solutions are highly dependent on a reliable bond between the different materials [20]. Cementation can be done using either conventional or adhesive techniques. The conventional technique requires mechanical retention *via* a sufficient retention cylinder and a surface roughness that creates micromechanical retention [8,9,21–25]. The adhesive technology requires chemical and micro-mechanical retention. To achieve a reliable and durable bond between a titanium base and a polymer-based material, adhesive cement systems in combination with some kind of pre-treatment of the cementation surfaces are usually recommended [21–25]. In addition, the mechanical retention can be improved by the design and the surface roughness of the titanium base [8,9,21]. There are a large number of different types of cements recommended for titanium and polymer-based materials and, additionally, new polymer-based materials are emerging, causing an issue for the dental teams when choosing the most appropriate cement system for achieving a strong and durable bond [7,26]. Furthermore, the recommendations of for example the pre-treatment of the cementation surfaces can differ between the cement and material manufacturers [11]. The requirements for the materials included in the present study were various polymer-based materials for temporary implant-supported FDPs and cements with the ability to bond to titanium. The aim of the study was to evaluate the bond strength between titanium used for titanium bases and polymer-based materials for prosthetic restorations cemented with different adhesive cement systems. The null hypothesis is that, independent of cement system, there will be no differences in bond strength between the titanium and the polymer-based materials.

## Materials and methods

According to a power analysis with 90% strength, significance level *α* = 0.05 and a significant difference between groups of 10 N, 13 specimens were included in each group. Each specimen consisted of two parts, a cylinder of titanium and a cylinder of polymer-based material. The titanium cylinders (Titan Grad 5, Elos Medtech, DK-3330 Gorlose, Denmark), representing a titanium base in an implant system, were manufactured with the dimensions 10 mm in diameter and 10 mm in height. The polymer-based cylinders with the dimensions 5 mm in diameter and 3 mm in height were designed in Meshmixer. The files were converted to STL files and sent to KaVo CAM2 (Kavo Dental gmbH, 88400 Biberach, Germany) for milling and NextDent 5100 3 D Printer (NextDent B.V., 3769 AV Soesterberg, Netherlands) for 3 D printing. In total, 104 cylinders of titanium and 52 cylinders of the material Telio CAD (Telio CAD, A16, Lot: VP1553, Ivoclar Vivadent, Schaan, Liechtenstein) were milled, and 52 cylinders of the material Micro Filled Hybrid, MFH (Micro Filled Hybrid, NextDent B.V., 3769 AV Soesterberg, Netherlands) were 3D printed. The materials were divided into groups according to the four different cement systems used. An overview of the materials, material content and the different groups with abbreviations is summarised in Table 1.

**Table 1.** The materials used in the study, including main material content and the abbreviation of each group. Grey zone: the polymer-based material, white zone: the different adhesive cement systems.

Materials	Main material content	Groups	
Polymer; Micro Filled Hybrid (MFH)	Components: Monomer based on acrylic esters (methacrylic oligomer >60%, glycol methacrylate 15–25%, phosphine oxide >2.5%), inorganic fillers		
Polymer; Telio CAD (Telio)	Components: Polymethyl methacrylate (PMMA), pigments		
Multilink Hybrid Abutment (Multi) including SR Connect	Dual cure Components: Dimethacrylate, HEMA, fillers (barium glass, ytterbium trifluoride, spheroid mixed oxides, titanium dioxide) Components: Methyl methacrylate, polymethyl methacrylate, dimethacrylates, initiators	T-Multi-MFH	T-Multi-Telio
Panavia V5 (V5) including Clearfil Ceramic Primer Plus	Dual cure Components: Bisphenol A, diglycidyl methacrylate triethyleneglycol dimethacrylate, silanated barium glass filler, silanated fluoroalminosilicate glass filler colloidal silica, aluminum oxide filler, hydrophobic aromatic dimethacrylate, hydrophilic aliphatic dimethacrylate dl-Camphorquinone, initiators accelerators, pigments Components: Original MDP adhesive monomer, y-MPS silane monomer	T-V5-MFH	T-V5-Telio
RelyX Ultimate Adhesive Resin Cement (RelyX) including Scotchbond Univeral Adhesive Primer	Dual cure Components: Methacrylate monomers, methacrylate monomers radiopaque, silanated fillers radiopaque alkaline (basic) fillers, initiator components, stabilizers, rheological additives, pigments, fluorescence dye Components: MDP phosphate monomer dimethacrylate resins HEMA Vitrebond™ copolymer, filler, ethanol, water, initiators, silane	T-RelyX-MFH	T-RelyX-Telio
G-CEM-LinkAce (GCem)	Dual cure Components: Urethane dimethacrylate (UDMA) 10–20%, dimethacrylate 10–20%, 2-hydroxy-1,3 dimethacryloxypropane 5–10%, methacryloyloxydecyl dihydrogen phosphate 2.5–5%, surface-treated silica, silane, synergist, α,α-dimethylbenzyl hydroperoxide <1%, 6-tert-butyl-2,4-xylenol <0.25%	T-GCem-MFH	T-GCem-Telio

Main material content is supplied by the manufacturers.

### Pre-treatment

Each specimen consisted of titanium-cement-polymer-based material. The cementation surface of the titanium cylinder was sandblasted with 50 µm AlO_2_ (Sandblaster Basic Quattro, Sandblasting medium Cobra, Precious Corundum, Renfert GmbH, Hilzingen, Germany) at 2 bar pressure. The distance between the nozzle of the sandblaster and the titanium surface was 10 mm and each surface was blasted for 5 s with the nozzle fixed straight to the surface being blasted. Thereafter, each surface was steam blasted for 5 s at a 50 mm distance. Each titanium cylinder was cemented to a cylinder of polymer-based material immediately after the steam blasting. The cementation surface of the MFH cylinders was also sandblasted according to the same procedure as for the titanium surfaces.

### Cementation

#### Cementation of T-Multi-Telio and T-Multi-MFH

Monobond Plus (Monobond Plus, Lot: X49303, Ivoclar Vivadent, Schaan, Liechtenstein) was applied to the cementation surface of the titanium and allowed to react for 60 s. After the reaction time, the excess was rinsed off with water and the surface was airblasted for 5 s. The cylinders of Telio CAD and MFH were steam blasted and air-dried with the air blaster for 5 s, according to the manufacturer’s instructions. The cementation surface was covered with SR Connect (SR Connect, Lot: WL4001, Ivoclar Vivadent, Schaan, Liechtenstein) using a disposable brush. After 30 s, light-curing with Ivoclar Bluephase Style 5 V (Bluephase Style 5 V, S/N:1100019329, Ivoclar Vivadent, Schaan, Liechtenstein) for 40 s followed. With the Multilink Hybrid Abutment (Multilink Hybrid Abutment HO 0, Lot: X46615, Ivoclar Vivadent, Schaan, Liechtenstein) syringe, a thin layer of cement was applied to the polymer-based surface. The titanium cylinder and Telio CAD or MFH were cemented under a constant pressure of 15 Newton (N) in order to standardise the cementation pressure in the process while checking that cement excess was present on all sides. The excess was removed with a disposable brush and each side light-cured for 20 s, from four different directions, 90 degrees apart. The load was removed from the specimen and light-cured for an additional 60 s to finally self-cure for another 7 min.

#### Cementation of T-V5-Telio and T-V5-MFH

K-Etchant (K-Etchant Syringe, Lot: 2Q0056, Kuraray Noritake, Co., Ltd. Kurashiki, Japan) was applied to the surfaces to be cemented and allowed to function for 5 s, followed by water rinsing for 10 s and air-blasting for 5 s. Thereafter, Clearfil Ceramic Primer Plus (Clearfil Ceramic Primer Plus, Lot: 1W0023, Kuraray Noritake, Co., Ltd. Kurashiki, Japan) was brushed on the titanium and Telio CAD or MFH with a disposable brush and the surfaces were air blasted for 5 s. With the mixing tip, Panavia V5 Paste (Panavia V5 Paste Lot: 1R0010, Kuraray Noritake, Co., Ltd. Kurashiki, Japan) was applied to the polymer-based cementation surface. The surfaces were cemented together under constant pressure of 15 N, cement excess was removed, and all surfaces were light-cured for 20 s from each four sides. The specimen was removed and light-cured from above for an additional 60 s to finally self-cure for 3 min.

#### Cementation of T-RelyX-Telio and T-RelyX-MFH

The surfaces were washed with ethanol and self-dried. 3 M ESPE Scotchbond Univeral Adhesive Primer (3 M ESPE Scotchbond Universal Adhesive, Lot: 90206 C, St. Paul, MN, USA) was applied to the titanium and allowed to function for 20 s. 3 M RelyX Ultimate Paste (RelyX Ultimate Paste, Lot: 4266223, St. Paul, MN, USA) was applied to Telio CAD or MFH and the surfaces were cemented together under pressure of 15 N, the cement excess was removed, and all surfaces were light-cured for 20 s from each four sides. The specimen was removed and light-cured from above for an additional 60 s, to finally self-cure for another 6 min.

#### Cementation of T-GCem-Telio and T-GCem-MFH

The surfaces of the titanium cylinder and Telio CAD or MFH were cemented together with G-Cem LinkAce **(**G-Cem LinkAce^®^, Lot: 1806262, GC Corporation, Tokyo, Japan) under constant pressure of 15 N, the cement excess was removed, and all surfaces were light-cured for 20 s from each four sides. The specimen was removed and light-cured from above for an additional 60 s, to finally self-cure for another 4 min.

#### Thermocycling

After cementation, each specimen was placed in distilled water and stored at 35 °C for 24 h before the specimens were thermocycled (Thermocycler 1100/1200, SD Mechatronik, Germany) for 5000 cycles to stress the interfaces between the different materials [27]. A cycle lasted for a total of 60 s: 20 s in two baths with temperatures of 5 °C and 55 °C with 10 s transfer time between the baths.

#### Shear bond strength test

The shear bond strength test to evaluate the bond strength was performed with a universal testing machine, Instron (Type 4465, Instron, S/N:4465H2276, Canton, MA, USA) with a loading speed of 0.5 mm/min. The load was placed between the titanium cylinder and the polymer-based material. The load at failure was measured in Newtons (N) and converted to megapascals (MPa) by the formula N divided by the cementation area.

### Statistical analysis

The data were analysed with one-way ANOVA, *post hoc* Tukey's test (software IBM SPSS, version 25.0, Armonk, NY: IBM Corp, USA) with a significant level of *α* = 0.05.

### Fracture analysis

The fractures were categorised into adhesive or mixed fractures. A fracture was classified as an adhesive fracture when more than 95% remnant of the cement was left on either the polymer-based surface or the titanium surface. For mixed fractures, both cohesive and adhesive fractures were required on both surfaces. The assessments were performed using a microscope (Wild M3, M7A Wild Heerbrugg, Heerbrugg, Switzerland) and both cementation surfaces of each specimen were photographed.

## Results

### Bond strength

Some of the specimens debonded through spontaneous bond failure (loss of retention) between the titanium-cement-polymer-based material during the water storage or during the thermocycling, before a shear bond strength test could be performed. The results are summarised in Table 2. In the groups with Telio CAD cemented with Panavia V5 (T-V5-Telio) and cemented with G-Cem LinkAce (T-GCem-Telio), 12 specimens in each group debonded during thermocycling. These two groups had high rates of debonding and a significantly lower bond strength compared to all other groups (*p* < .05). In the group T-Multi-Telio and in the group T-V5-MFH, three specimens debonded in each. The highest bond strength, although not significant, was found in the groups with MFH cemented with RelyX Ultimate (T-RelyX-MFH) and MFH cemented with G-Cem LinkAce (T-GCem-MFH). Within the Telio CAD groups, the cement RelyX Ultimate showed the highest bond strength, however, the Telio CAD groups generally showed lower bond strength values than the MFH groups.

**Table 2.** The results from the shear bond strength test in MPa (mean, SD, maximum and minimum), including number of specimens.

Groups	*n* for SBS-test (total *n*)	Mean, MPa	SD	Maximum, MPa	Minimum, MPa
T-Multi-Telio	10 (13)	7	3.7	15.4	0.0
T-V5-Telio	1 (13)	0.06^a^	0.05^a^	0.8^a^	0.0^a^
T-RelyX-Telio	13 (13)	20	2.7	28.8	14.7
T-GCem-Telio	1 (13)	0.08^a^	0.09^a^	1.07^a^	0.0^a^
T-Multi-MFH	13 (13)	24	10.8	35.7	3.0
T-V5-MFH	10 (13)	11	10.6	30.2	0.0
T-RelyX-MFH	13 (13)	33	2.5	42.1	22.9
T-GCem-MFH	13 (13)	32	4.7	42.9	12.4

^a^The majority of the specimens debonded (loss of retention) before shear bond strength testing.

### Fractures

**Figure 1.**
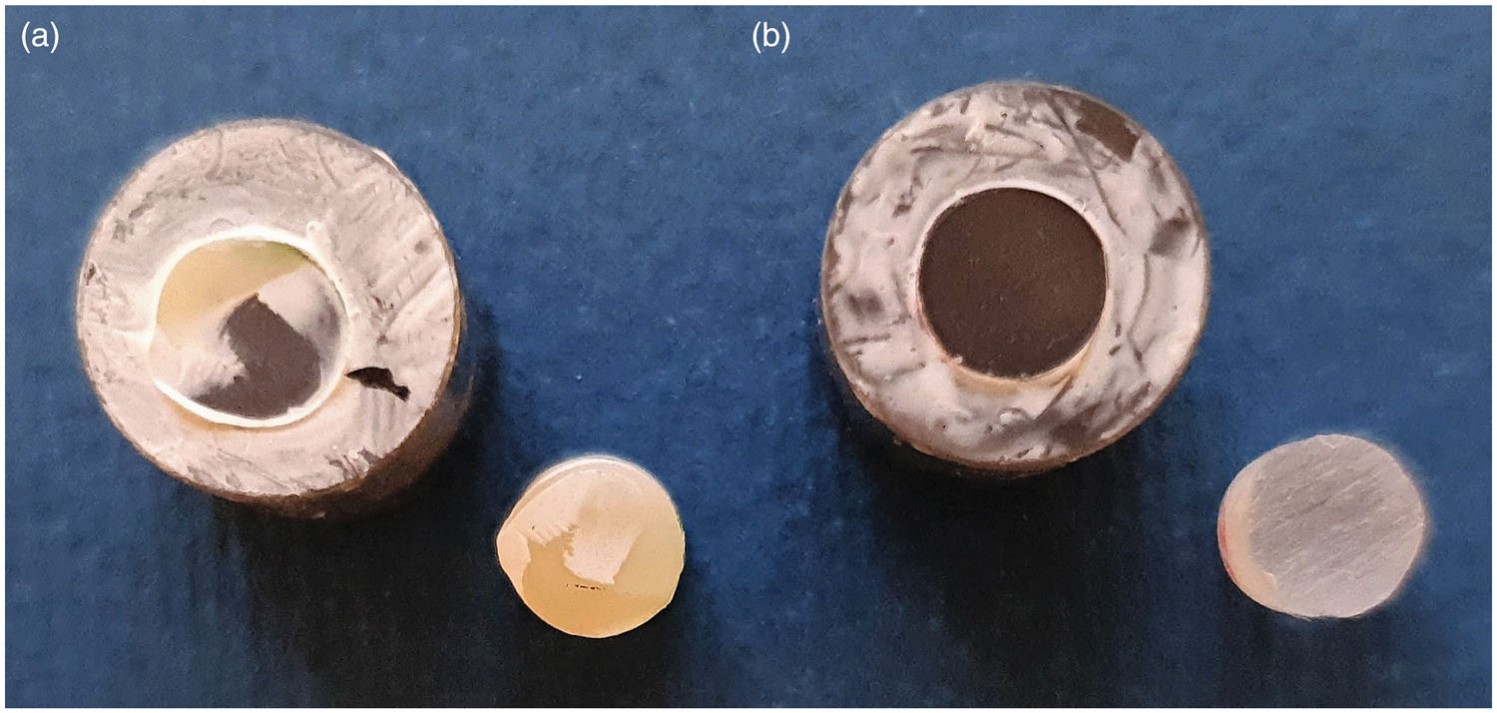
Representative images of adhesive and mixed fractures. (a) Mixed fracture for group T-Multi-MFH. (b) Adhesive fracture for group T-GCem-Telio. The cement remains only on the cementation surface of the titanium and has completely detached from Telio CAD. The white areas on the titanium surface is the thin layer of primer from the respective cement systems.

The fracture types differed in comparison between the groups, summarised in Table 3. The distribution of fracture types among the groups with MFH were dispersed, including adhesive fractures with cement residues on the cementation surface of the titanium and on the cementation surface of the MFH as well as mixed fractures with cement residues on both the titanium and MFH, Figure 1(a). For all specimens with Telio CAD, there were pure adhesive fractures with the cement remaining on the titanium surface, Figure 1(b).

**Table 3.** Distribution of type of fracture (adhesive and mixed failure) per group.

Groups	*n* for SBS-test (total *n*)	Adhesive (*n*)	Mixed (*n*)
T-Multi-Telio	10 (13)	13	0
T-V5-Telio	1 (13)	13	0
T-RelyX-Telio	13 (13)	13	0
T-GCem-Telio	1 (13)	13	0
T-Multi-MFH	13 (13)	6	7
T-V5-MFH	10 (13)	6	7
T-RelyX-MFH	13 (13)	3	10
T-GCem-MFH	13 (13)	13	0

## Discussion

The results summarised in the present study show a high rate of debonding where the polymer cylinder lost retention to the titanium cylinder during the first 24 h of water storage, and some during the following thermocycling. The bond strength between Panavia V5-Telio and G-Cem LinkAce-Telio was the lowest, as the majority lost retention before or during thermocycling, and are not sufficient for relying on an adhesive bond between titanium and polymer-based material. Thermocycling is a well-used method for simulating the stresses and aging a material is exposed to in the oral cavity, preventing unrealistic values, and is especially suitable for stressing interfaces [28], thus all groups were subjected to thermocycling. It has been shown that the ability of polymers to absorb water can affect their properties and the temperature differences can cause volume changes, creating stresses and microcracks in the interfaces between the materials [29]. Inclusion of non-thermocycled groups might have provided additional information to analyse the mechanism of the failures. Nevertheless, restorations are required to be able to withstand temperature changes in the oral cavity.

The bond strength of the MFH groups was higher than the Telio CAD groups, and only two cylinders lost retention during the thermocycling. Overall, 3 M RelyX Ultimate was the cement system that showed a relatively high bond strength to both polymer-based materials, although G-Cem LinkAce and Multilink Hybrid Abutment had higher bond strength to MFH than Rely X Ultimate to Telio. The bond strength values for these groups (T-RelyX-Telio, T-Multi-MFH, T-RelyX-MFH, T-GCem-MFH) are adequate for provisional cementation. Dhesi et al. evaluated the shear bond strength between sandblasted titanium and etched ceramic or etched hybrid materials with different cements [30]. They reported that the bond strength after thermocycling for the hybrid material Lava Ultimate was 1.16 MPa with Panavia 21 and 15.1 MPa with Multilink Hybrid Abutment and for Vita Enamic 12.4 MPa and 24.9 MPa for the corresponding cements. Even if Dhesi et al. included hybrid materials that per definition have a larger amount of ceramic structure and are etchable, similar results are shown in the present study, where the bond strength for Telio cemented with Panavia V5 was 0.06 MPa and with Multilink Hybrid Abutment 7 MPa, and for MFH 11 MPa and 24 MPa for the corresponding cements. These findings may be due to various factors, such as the different content of the materials, cement systems and the different pre-treatments performed. When hybrid materials are pre-treated through etching, the surface roughness is increased and the ceramic and cement system facilitate a chemical reaction [30]. Materials used in the present study are polymethyl methacrylate (PMMA)-based (Telio) and a hybrid material with polymer esters and a high content of inorganic fillers (MFH), and are not recommended for etching.

The manufacturers of the respective materials could sometimes recommend different types of pre-treatment. The surface of the titanium was chosen to be sandblasted, creating a micro-mechanical surface, but the cementation surface of Telio CAD was only pre-treated with SR Connect, as recommended by the manufacturer for the polymer-based material. As MFH is a relatively new material, it was difficult to access the manufacturer's recommendations and pre-treatment of the cementation surface, thus it was carried out according to the cement manufacturers' recommendations, which involved sandblasting of the cementation surfaces of the specimens made of MFH. In previous studies, it has been established that sandblasting in combination with different metal primers results in a higher bond strength between titanium and PMMA [9,30,31].

In the Telio CAD groups, all cylinders had adhesive failures, indicating that the bond between the different adhesive cement systems and Telio CAD is unstable. On the contrary, MFH showed a stronger bond to the cement since the failures were a mixture of cohesive and adhesive fractures indicating a higher probability that the temporary restoration will last until the permanent restoration is to be cemented. The adhesive fractures of the Telio CAD groups indicate that the chemical interaction with all tested cements except RelyX Ultimate is insufficient, and some type of abrasive surface treatment is needed. The manufacturer of Telio CAD recommends the application of SR Connect to the cementation surface of Telio CAD prior to cementation with Multilink Hybrid Abutment. However, some specimens debonded before the shear bond strength test, indicating that SR Connect without sandblasting results in lower bond strength. The findings in the present study highlight the importance of pre-treatment methods, but further studies are required to confirm this assumption. In accordance, the instructions for use for Telio CAD, published after the study was conducted, have now been updated to include sandblasting of the cementation surface.

The requirement for the cements included in the study was their ability to bond to titanium. All cement systems were treated similarly regarding the curing process, i.e. dual cured, however, the content of the systems differed. Multilink is a dimethacrylate- and HEMA-based adhesive cement system that requires the use of a primer such as SR Connect. The Panavia V5 cement system includes primers containing phosphate monomers (MDP) and is primarily developed for metals or ceramic-based restorations. The RelyX Ultimate cement system includes a primer, Scotchbond, that contains phosphate monomers, dimethacrylate and HEMA, making the cement system more stable and compatible with different materials. G-CEM-LinkAce consists of urethane dimethacrylate (UDMA) and dimethacrylate. The chemistry of the cements in combination with the properties of the respective polymer-based material might partly explain the results between and within groups, e.g. the bond between Panavia V5 with the MDP primer and PMMA-based materials, as Telio CAD might be less stable, hence more susceptible to thermocycling than the bond to a hybrid material, due to the inorganic filler content. A chemical analysis of the materials was however not conducted and could be included in future studies.

The specimens were designed with flat surfaces, in order to evaluate the micromechanical and chemical retention, reducing the possible influence of the geometry of an abutment or a restoration. More complex specimens subjected to chewing force could be considered in future studies. The height of the cylinder was based on the chosen test method. An excessively high cylinder can result in tension stresses rather than shear forces, thus the strength of the cylinder and not the interface between titanium-cement-polymer-based materials is evaluated. It is also important that the cementation surfaces and the height of the cylinders are equal in order to obtain an even pressure during the cementation, thus an evenly thick cement layer [32]. The dimensions and flatness were controlled, but small deviations cannot be ruled out, possibly resulting in a non-uniformly applied pressure to the specimens during the cementation. This, in turn, can lead to a slight difference in the thickness of the cement layer, affecting the results, and is a limitation of the present study due to the absence of a cement thickness evaluation.

Based on the results of the study, the null hypothesis can be rejected, as there are differences between the values of the bond strength between titanium-adhesive cement systems-polymer-based materials.

## Conclusion

Based on the results of the study and with regard to the limitations, the following conclusions can be drawn: pre-treatment methods and choice of cement system are of importance for polymer-based materials for prosthetic restorations. The bond strength is adequate for provisional cementation irrespective of cement system when pre-treating by sandblasting, but cement dependent without sandblasting.
